# A case of the effective inhalation of nitric oxide therapy for caused severe pulmonary hypertension with protamine neutralization of systemic heparinization during totally endoscopic minimally invasive cardiac surgery

**DOI:** 10.1051/ject/2024018

**Published:** 2024-09-20

**Authors:** Tomohisa Takeichi, Yoshihisa Morimoto, Akitoshi Yamada, Takanori Tanaka

**Affiliations:** 1 Department of Clinical Engineering, Kitaharima Medical Center 926-250, Ichiba-cho Ono-shi Hyogo 675-1392 Japan; 2 Department of Cardiovascular Surgery, Kitaharima Medical Center 926-250, Ichiba-cho Ono-shi Hyogo 675-1392 Japan

**Keywords:** Cardiopulmonary bypass (CPB), Nitric oxide, Respiratory therapy, Pulmonary hypertension, Protamine

## Abstract

Severe pulmonary vasoconstriction induced by protamine is a rare complication. We report a case of a 77-year-old male patient with a history of mitral valve plasty (MVP). He underwent redo MVP via right thoracotomy under the totally endoscopic procedure (MICS redo-MVP). Immediately after weaning cardiopulmonary bypass (CPB), protamine was administrated. 10 min later peak systolic pulmonary arterial pressure (sys PAP) rose to 62 mmHg, and 30 min later to 80 mmHg. Due to the negative impact of protamine administration, nitric oxide inhalation (iNO) therapy was started with a concentration of 20 ppm. 10 min after iNO therapy started, sys PAP decreased to 63 mmHg. After entering the intensive care unit (ICU), sys PAP decreased to 35 mmHg. Here, we present an effective iNO therapy case for pulmonary hypertension due to protamine and the patient had a good postoperative recovery.

This study was approved by the Institutional Review Board at Kitaharima Medical Center (IRB-0602) with the waiver of informed consent.

## Introduction

Protamine covalently binds to anionic heparin forming a stable precipitate and neutralizing the anticoagulant effect of heparin [1, 2]. On the other hand, there are some adverse effects such as Type I, characterized by hypotension due to rapid infusion; Type II, presenting as an anaphylactic-like reaction; and Type III, associated with severe pulmonary hypertension caused by significant pulmonary vasoconstriction [1, 3–7]. Notably, Type III (catastrophic pulmonary hypertension) is rare, and often results in right heart failure. There are not many reports using iNO therapy for pulmonary hypertension of protamine adverse effects [2, 8, 9]. In this case report, we report a patient who developed a marked elevation of sys PAP (80 mmHg) following protamine administration, but without evidence of RV (right ventricular) failure or systemic hypotension, for which iNO therapy was started at a concentration of 20 ppm. PAP gradually decreased, and after entering the ICU, sys PAP decreased to 35 mmHg. iNO therapy was started with a concentration of 20 ppm, PAP gradually decreased, and after entering the ICU, sys PAP decreased to 35 mmHg.

## Case report

The patient (height 155 cm; weight 64.1 kg) had a history of MVP twenty-three years ago. The patient was diagnosed with moderate mitral valve regurgitation (MR) recurring by transthoracic echocardiography (TTE). Additional echo findings were left ventricular ejection fraction (LVEF) of 56%, left ventricular internal dimension in diastole (LVDd) of 56.4 mm, left ventricular internal in systole (LVDs) was measured at 39.5 mm, tricuspid regurgitation peak gradient (TRPG) of 42.5 mmHg, and tricuspid valve regurgitation (TR). A right heart catheterization revealed features indicative of moderate pulmonary hypertension (PH) with the following parametric values: PAP, 52/23 mmHg (mean, 30 mmHg), pulmonary capillary wedge pressure (PCWP), 24 mmHg, RVP 38/12 (mean, 15 mmHg), central venous pressure (CVP), 17 mmHg, cardiac index (CI), 2.8 L/min/m^2^, calculated total pulmonary resistance (TPR), 1733 dyne/s/cm^5^, and pulmonary vascular resistance (PVR), 552 dyne/s/cm^5^. We planned to redo MVP under totally endoscopic. EuroSCORE II was 11.9.

**Figure 1:**
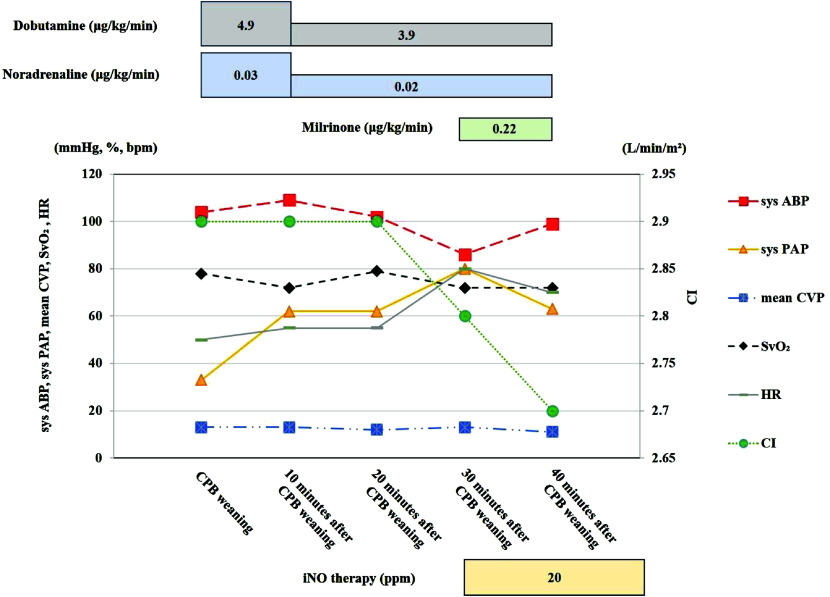
The figure of after weaning CPB course. Sys ABP.: Systolic arterial blood pressure; Sys PAP.: Systolic pulmonary artery pressure; mean CVP.: Mean central venous pressure; SvO_2_.: Heart rate; HR.: Mixed venous oxygen saturation; CI.: Cardiac index; iNO.: Inhaled nitric oxide.

Following induction of general anesthesia, a pulmonary artery (PA) catheter (Swan-Ganz CCOmbo model: 744HF75, Edwards(r), USA) was inserted via an introducer (8.5 Fr) placed in the right internal jugular vein. The patient underwent MICS redo MVP. CPB was established with a venous cannula 23/25Fr (MICS Cannulae; LivaNova, Tokyo, Japan) placed in the right femoral vein and an arterial cannula 18Fr (PCKC-A, MERA, Tokyo, Japan) placed in the right femoral artery. Centrifugal pump (MERA Centrifugal Pump HCF-MP23, SENKO MEDICAL INSTRUMENT, Inc., Tokyo, Japan) was used for CPB, with a target pump flow was 2.4 L/min per m^2^. Phenylephrine and noradrenaline were administered to maintain a mean arterial pressure above 60 mmHg. Anticoagulation was given at an initial dose of 250 IU/kg (15000 IU) to achieve a target activated clotting time of at least 480 s and if the activated clotting time was less than 480 s, an additional dose of 4000 IU was given. A CDI Blood Parameter Monitoring System 500 (Terumo, Tokyo, Japan) was recalibrated every 30 min, and an arterial blood gas sample was also checked every 30 min. The patient was cooled to 31 °C. Cardiac arrest was achieved using antegrade cardioplegia, and the mitral valve approach exposure was performed through the left atrium. Redo MVP was carried out as planned. Weaning from CPB was performed using dobutamine 0.03 ug/kg/min and noradrenaline 4.9 ug/kg/min (Fig. 1). CPB time and aortic cross-clamp time were 235 min, and 116 min, respectively. Immediately after weaning CPB, protamine was administrated dose of 13 mg. Protamine was administrated over 10 min from the peripheral venous route by dripping. At the same time, the dose of dobutamine and noradrenaline was decreased to 0.02 ug/kg/min and 3.9 ug/kg/min, respectively. Ten minutes after CPB weaning, sys PAP increased from 33 mmHg to 62 mmHg. However, systolic arterial blood pressure (sys ABP), mixed venous oxygen saturation (SvO_2_), heart rate (HR), CI, and CVP were 109 mmHg, 72%, 50 bpm, 2.9 L/min/m^2^, and 13 mmHg, and no major changes were observed. Moreover, 30 min later, the sys PAP was 80 mmHg. Sys ABP decreased slightly, but, SvO_2_, HR, CI, and CVP were stable. Considering the adverse impact of protamine, iNO therapy was started with a concentration of 20 ppm. Also, the administration of milrinone was started at 0.22 ug/kg/min. Ten minutes after iNO therapy was started, sys PAP decreased to 63 mmHg, while, sys ABP increased to 99 mmHg. SvO_2_, HR, CI, and CVP did not change significantly, respectively. The operation time was 303 min, and the total fluid balance was 9096 mL.

**Figure 2:**
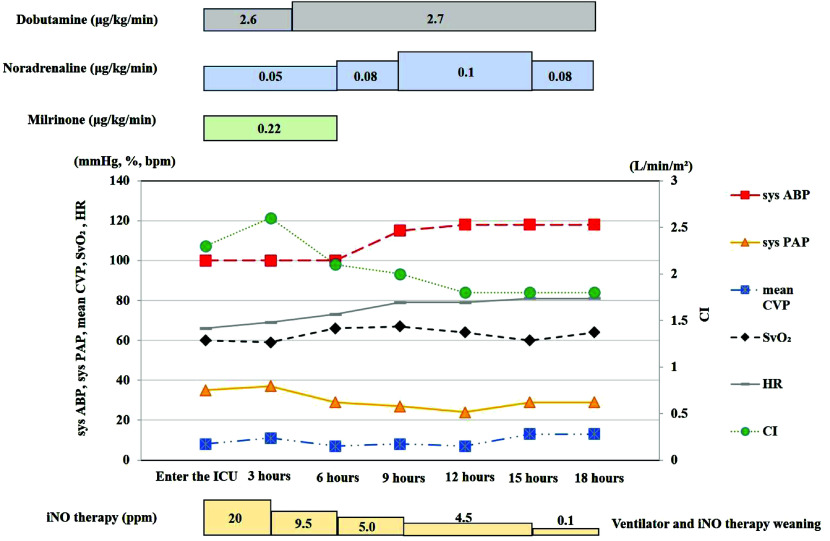
The figure after entering the ICU course. Sys ABP.: Systolic arterial blood pressure; Sys PAP.: Systolic pulmonary artery pressure; mean CVP.: Mean central venous pressure; SvO_2_.: Heart rate; HR.: Mixed venous oxygen saturation; CI.: Cardiac index; iNO.: Inhaled nitric oxide.

**Figure 3:**
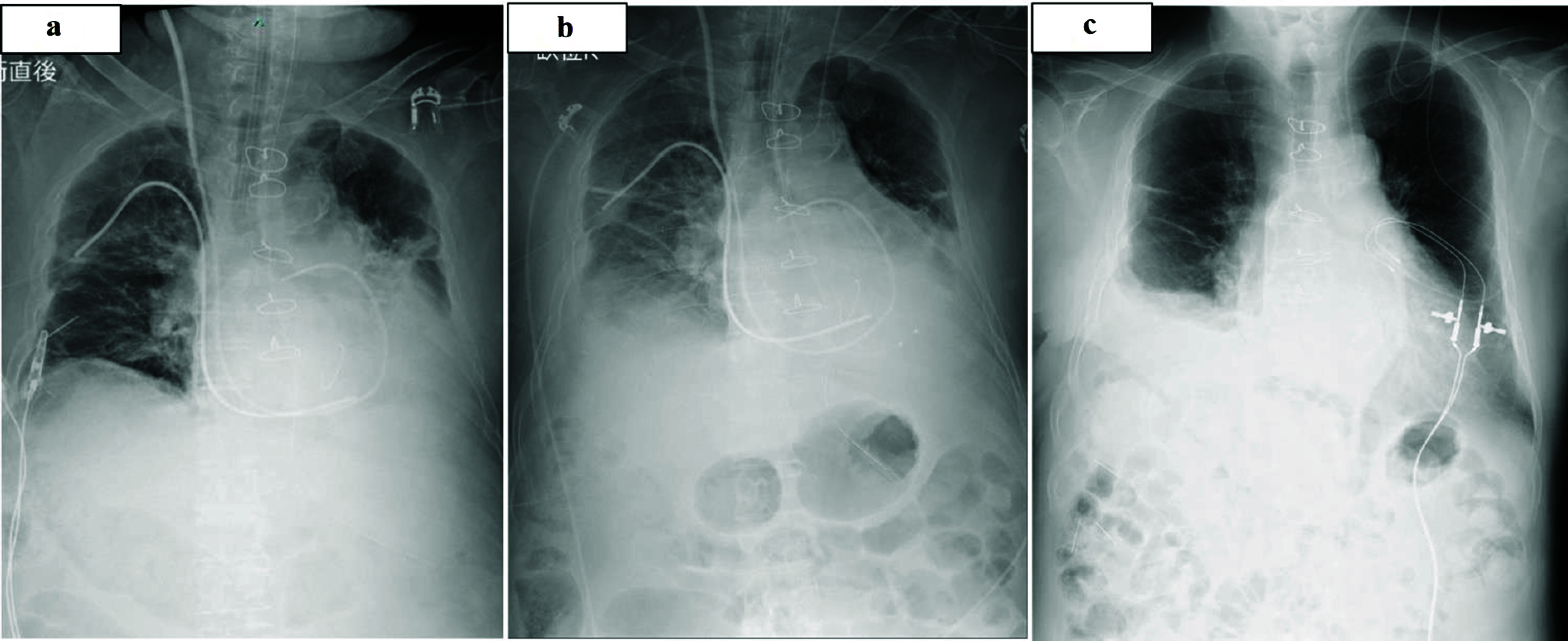
(a, b) Immediately postoperative CXR and the day after surgery CXR, which indicates overall pulmonary edema. (c) CXR indicates gradually improving from 5 days after surgery. CXR.: Chest x-ray.

After entering the ICU, sys PAP decreased to 35 mmHg, and sys ABP was 100 mmHg. The parameters such as SvO_2_, HR, CI, and CVP were also stable (Fig. 2). P/F (PaO_2_: 109 mmHg, FiO_2_: 0.5) ratio was 218. The administration of dobutamine was 2.6 ug/kg/min, noradrenaline was 0.05 ug/kg/min, and milrinone was 0.22 ug/kg/min. After 3 h of entering the ICU, iNO concentration was gradually reduced, and after 18 h, iNO therapy was stopped, and extubation could be performed. A postoperative chest x-ray (CXR) was revealed (Fig. 3a–c). Overall pulmonary edema appeared immediately postoperative and the day after surgery (Fig. 3a, b). 5 days after surgery, CXR was gradually improved (Fig. 3c). Length of stay in the ICU was 2 days. The postoperative course was uneventful and the patient was discharged after undergoing antibiotic treatment for 2 weeks.

Informed consent to report patient information and images was obtained.

## Discussion

Protamine covalently binds to anionic heparin forming a stable precipitate and neutralizing the anticoagulant effect of heparin, and is routinely used in cardiac surgery employing CPB [1, 10]. The adverse effects of protamine include hypotension, arrhythmias, diaphoresis, flushing, altered consciousness level, catastrophic pulmonary hypertension, and anaphylaxis and anaphylactoid reactions. The diagnosis of this complication is often empirical, based on its close temporal proximity to protamine administration after excluding other possible causes. In patients not receiving Neutral Protamine Hagedorn (NPH) insulin and protamine-zinc insulin (PZI), the incidence of hypotensive adverse reactions is reported to be 0.06%, whereas anaphylaxis-like reactions (severe hypotensive reactions) in patients receiving NPH insulin are shown to be 0.6% [1, 10, 11]. Also, the most severe and persistent adverse response to protamine administration for heparin reversal seems to be an idiosyncratic reaction that may be related to previous exposure to protamine [12, 13]. On the other hand, Levy, et al concluded that prior NPH insulin use, a history of fish allergy, or prior vasectomy does not represent a contraindication to protamine administration after CPB [4]. In our case, the patient had no history of NPH insulin and PZI use. Following protamine administration over 10 min from the peripheral venous route, sys PAP increased to 80 mmHg, prompting initiation of iNO therapy, resulting in a subsequent decrease in sys PAP experienced by the patients. The adverse reactions to protamine administration are classified into three types: Type I, characterized by hypotension due to rapid infusion; Type II, presenting as an anaphylactic-like reaction; and Type III, associated with severe pulmonary hypertension caused by significant pulmonary vasoconstriction. Type II reactions are further subclassified into a) immediate-type anaphylactic reactions, b) immediate-type anaphylactic-like reactions, and c) delayed-type anaphylactic-like reactions [1, 3]. In our case, because of the increased sys PAP, followed soon after protamine administration, a type III protamine reaction was suspected. Pulmonary hypertension due to severe pulmonary vasoconstriction of type III can rapidly occur following protamine administration [1, 3]. As a result of starting with iNO therapy, sys PAP gradually decreased. Fortunately, hemodynamics such as sys ABP was no major change. After admission to the ICU, sys PAP was a normal value. More often, this catastrophic pulmonary hypertension results in right ventricular failure, low cardiac output, and systemic arterial hypotension, and interventions such as inhaled NO or epoprostenol, and even veno-arterial extracorporeal membrane oxygenation (V-A ECMO) are required [2, 14]. Also, to decrease PAP, Zheng Guan et al showed the effectiveness use of epoprostenol to treat severe pulmonary vasoconstriction [15–17]. Heparin reversal by protamine has been reported to cause leukocytosis, and increased levels of complement factor C5b‑9, interleukin (IL)‑6, and IL‑8 [18]. Our patient experienced not only pulmonary hypertension but also pulmonary edema, possibly related to increased pulmonary vascular permeability, with a P/R ratio of 218. In the case of protamine type II reaction of delayed-type anaphylactic-like reactions, after 20–60 min of administration, this reaction is also observed along with increased pulmonary vascular permeability [19, 20]. However, a major contributor to the pulmonary edema in our patient was the excessive fluid balance; the total fluid balance was 9096 mL. We hypothesize that the reason this patient did not experience right heart failure and systemic hypotension was that this patient had chronic pulmonary hypertension, and therefore, the right ventricle might have been prepared (“conditioned”) to tolerate this increased PAP. This is supported by MacNee W, et al. who reported that RV’s stroke volume is decreased with increased afterload compared with the left ventricle [21]. However, despite the RV’s thinner wall (2–5 mm, about one-sixth of the LV’s thickness), it achieves comparable stroke volume and CO to the LV. In pulmonary arterial hypertension (PAH), progressive pulmonary vascular disease increases PVR and RV afterload, causing RV wall stress and right ventricular hypertrophy (RVH). These changes maintain RV–PA coupling, allowing the RV to adapt and sustain stroke volume. However, with maximal RVH and rising PVR, the RV dilates to maintain CO, a maladaptive response that further increases wall stress [22, 23]. As a result, RV failure could be caused.

## Data Availability

All available data are incorporated into the article.
